# Fruit and vegetable intake and mental health among family caregivers of people with dementia in Uganda

**DOI:** 10.1016/j.mhp.2021.200223

**Published:** 2021-12

**Authors:** Herbert E. Ainamani, Wilson M. Bamwerinde, Godfrey Z. Rukundo, Sam Tumwesigire, Valence Mfitumukiza, Everd M. Bikaitwoha, Alexander C. Tsai

**Affiliations:** a Department of Mental Health, Kabale University School of Medicine, Kabale, Uganda, P. O. Box 317; b Department of Environment and Natural Resources Kabale University, Kabale, Uganda; c Department of Psychiatry, Mbarara University of Science and Technology, Mbarara, Uganda; d Department of Pediatrics, Kabale University School of Medicine, Kabale, Uganda; e Department of Nursing, Kabale University School of Medicine, Kabale, Uganda; f Center for Global Health and Mongan Institute, Massachusetts General Hospital, Boston, United States

**Keywords:** Anxiety, Dementia, Depression, Diet, Mental health, Stress, Sub-Saharan Africa, Uganda

## Abstract

Consumption of fruits and vegetables is correlated with improved mental wellbeing. Although this growing body of research has been recognized by researchers and clinicians in high-income countries, fewer studies examining this relationship have been conducted in low- and middle-income settings. In this study, we sought to estimate the association between fruit and vegetable intake and symptoms of depression and anxiety. We conducted a cross-sectional study among 242 family caregivers of people with dementia in southwestern Uganda. Fruit and vegetable intake in the past week was measured with a food frequency questionnaire. Depression and anxiety were assessed using the depression and anxiety subscales of the 42-item Depression, Anxiety and Stress Scales. Multivariable regression models were used to estimate the associations between fruits and vegetable consumption and depression and anxiety, adjusting for caregiving burden and other potential confounders. Depression symptom severity was negatively associated with consumption of jackfruits (b =-4.68; 95% confidence interval [CI], -8.96 to -0.39), green leafy vegetables (b =-14.1; 95% CI, -18.0 to -10.1), root vegetables (b =-14.0; 95% CI, -19.5 to -8.63), and other vegetables (b =-14.8; 95% CI, -19.3 to -10.3), and frequent consumption of vegetables (b =-1.91; 95% CI, -3.77 to -0.04). Anxiety symptom severity was negatively associated with consumption of green leafy vegetables (b =-12.2; 95% CI, -16.0 to -8.46), root vegetables (b=-12.6; 95% CI, -17.5 to -7.58), and other vegetables (b =-12.7; 95% CI, -17.0 to -8.40), and frequent consumption of vegetables (b =-2.07; 95% CI, -3.84 to -0.29). Our results suggest that fruit and vegetable consumption is associated with reduced depression and anxiety symptoms.

## 1 Background

Depression and anxiety are the most commonly studied mental health problems among the caregivers of older-age people with dementia (Alfakhri et al., 2018; Clyburn, Stones, Hadjistavropoulos, & Tuokko, 2000; Omranifard, Haghighizadeh, & Akouchekian, 2018), people with stroke (Alfakhri et al., 2018; Atteih et al., 2015; Balhara, Verma, Sharma, & Mathur, 2012; Guo & Liu, 2015) and those with mental ill health (Alqahtani et al., 2018; Minichil, Getinet, Derajew, & Seid, 2019). Studies on dementia report that, in addition to experiencing high levels of caregiving burden, caregivers of older-age people with Alzheimer's disease and other related dementias (ADRD) tend to have symptoms of depression and anxiety (Clyburn et al., 2000; Dotchin et al., 2014). A meta-analysis that included 17 studies on the prevalence of mental health disorders among caregivers of patients with Alzheimer's disease found that more than one-third had clinically significant symptoms of depression and anxiety (Sallim, Sayampanathan, Cuttilan, & Ho, 2015). In fact, the authors of the above meta-analysis observed that when compared with caregivers of other diseases, the prevalence of mental health problems among caregivers of people with dementia was higher than caregivers of other conditions.

Recent studies in nutrition and mental health, mostly from high income countries, suggest that consumption of some foods may be associated with improvements in mood (Gilbody, Lightfoot, & Sheldon, 2007; Li, Li, Song, & Zhang, 2017; McMartin, Jacka, & Colman, 2013; Péneau et al., 2011; Van der Does, 2001). For example, higher fruit and vegetable intake has been found to be associated with lower symptoms of depression and anxiety (Gibson-Smith et al., 2020; Liu, Yan, Li, & Zhang, 2016). In a cross-sectional study among 400 women with mental health problems attending health care centers, it was found out that participants in the lower quartile of fruit and vegetable intake were more likely to develop depression than those taking higher doses (Baharzadeh et al., 2018). Consistent with the above literature, a large meta-analysis of eighteen studies on fruit and vegetable intake observed a negative correlation between the consumption of fruits/vegetables and depression (Liu et al., 2016).

Although previous research studies in high-income countries have linked increased fruit and vegetable intake to lower levels of depression (McMartin et al., 2013; Tsai, Chang, & Chi, 2012) and anxiety (McMartin et al., 2013; Smith & Rogers, 2014), few studies about this phenomenon have been conducted in sub-Saharan Africa. One national survey of 3962 adult participants showed that the consumption of both fruits and vegetables in Uganda is low (Kabwama, Bahendeka, Wesonga, Mutungi, & Guwatudde, 2019). Yet the mental health benefits of fruit and vegetable consumption are well documented (Liu et al., 2016).

Given the significant role family caregivers play in the care of older-age persons with ADRD in Africa (Ainamani et al., 2020b; Kakongi et al., 2020; Kamoga et al., 2019; Mubangizi, Maling, Obua, & Tsai, 2020) and their high levels of symptoms depression and anxiety (Ainamani et al., 2020a; Ainamani et al., 2021), it is important to provide evidence on potentially modifiable factors that can reduce the mental health burden of depression and anxiety in this particular group of caregivers. Our study aimed to estimate the association between fruit and vegetable intake and symptoms of depression and anxiety in a sample of family caregivers of people with ADRD in a rural area of southwestern Uganda. We hypothesized that the consumption of different types of fruits and vegetables would be negatively correlated with symptoms of depression and anxiety among the caregivers of people living with dementia in rural, southwestern Uganda.

## 2 Methods

### 2.1 Population and design

We conducted a cross-sectional study to assess fruit and vegetable consumption and symptoms of depression and anxiety among 242 adult family caregivers of patients with ADRD in two districts of Kigezi, a rural region of southwestern Uganda. We collected data during July-August 2020. To be included in the study, participants had to be 18 years of age or older, identify as the family caregiver of someone living with ADRD, live in the same house or compound with the patient, and have provided care for more than 6 months. (In the remainder of this manuscript, for ease of exposition we refer to the caregivers as the “study participants” and to the family members with ADRD as their “patients.”) We sought guidance from the health workers of Reach One Touch One Ministries (ROTOM) (Rukiga District) and Heal Medical Centre (Rubanda District) to locate family caregivers of people with ADRD. We excluded caregivers who could not communicate to our study staff reliable information about their patients (e.g., due to deafness/mutism or acute intoxication). All interviews were conducted in Runyankore-Rukiga (the local language).

### 2.2 Ethical considerations

Approval to conduct this research was obtained from the Mbarara University of Science and Technology Research Ethics Committee, and clearance was obtained from the Uganda National Council for Science and Technology. Before the interview, information on the content, procedures, risks, the right to withdraw, and confidentiality were explained to prospective participants. Both oral and written informed consent were provided by each research participant at the beginning of every interview. Participants who did not know how to read and write were asked to provide a thumbprint for consent.

### 2.3 Primary exposures and outcomes of interest

Fruit and vegetable consumption was assessed using a dietary survey questionnaire (Brookie, Best, & Conner, 2018) that assesses the weekly consumption of different fruits and vegetables. For each fruit and vegetable type, study participants were asked the number of days per week of consumption (ranging from 0-7) and the number of servings consumed per day (ranging from 1-7 servings). Frequency of weekly milk consumption was assessed similarly. Specification of these variables was based on previous work (Baharzadeh et al., 2018; Choda et al., 2020; Crichton, Murphy, & Bryan, 2010).

The 42-item Depression, Anxiety and Stress Scales (DASS-42**)** was used to assess caregivers’ symptoms of depression and anxiety (Lovibond & Lovibond, 1995). This scale consists of depression, anxiety, and stress subscales, each containing 14 items. The items in each subscale are scored on a 4-point Likert-type scale (“never”, “sometimes”, “often”, “almost always”), yielding a total score ranging from 0 to 42 for each subscale, with higher scores indicating more severe symptoms of depression, anxiety, and stress. The DASS-42 has previously shown adequate internal consistency, with evidence of construct validity suggested by robust correlations with associated constructs (Ainamani et al., 2020a; Crawford & Henry, 2003; Gloster et al., 2008). In our sample, the Cronbach's α was 0.89.

### 2.4 Covariates

In addition to measuring the main explanatory variables, caregiving burden was assessed using the Zarit Burden Interview (ZBI) (Zarit, Reever, & Bach-Peterson, 1980). The ZBI is a 22-item scale that assesses various aspects of caregiving burden. The items are scored on a 5-point Likert scale that ranges from “never” to “nearly always,” with a total sum score that ranges between 0 and 88. This scale is commonly used in research studies of caregiving burden and its association with mental health problems (Ainamani et al., 2020a; Springate & Tremont, 2014). Patient functional status was measured using the Bristol Activities of Daily Living Scale (BADLS) (Bucks, Ashworth, Wilcock, & Siegfried, 1996). BADLS total scores range from 0 to 60, with higher scores indicating more severe functional impairment (Ainamani et al., 2020a; Jefferson et al., 2008). We also measured additional covariates: age and sex of both caregivers and their patients, the nature of the patients’ relationships with their caregivers, total years of caregiving, and caregivers’ educational attainment in years. Inclusion of these covariates was based on previous work linking them to mental health problems (Ainamani et al., 2020a; Kwon, Park, Kim, Choi, & Jang, 2019).

### 2.5 Data analysis

Using Stata software (version 16, Stata Corp., College Station, Tex.), we computed standard statistics to summarize characteristics of the sample. The association between fruit and vegetable consumption and mental health outcomes (depression and anxiety subscale scores specified as continuous variables) was estimated using multivariable linear regression models. To assess the robustness of our findings to potential confounding by unobserved variables, we conducted an e-value analysis (VanderWeele & Ding, 2017). The e-value estimates the minimum strength of association, on the risk ratio scale, that an unobserved confounder would need to have with both the exposure and the outcome in order to completely explain away the estimated association. A large e-value suggests that an unobserved variable would need to pose very strong confounding in order to invalidate the findings.

## 3 Results

### 3.1 Characteristics of the sample

The demographic characteristics are presented in Table 1. Out of 242 participants, most (75%) were women. Most of the participants (181 [75%]) were children of the patients, 36 (15%) were grandchildren, while 25 (10%) were spouses or others. Caregivers’ mean age was 44.4 years (standard deviation [SD], 14.8), and they provided care for a mean duration of 8.4 years (SD, 2.6). Caregiving burden was high, with a mean ZBI of 56.9 (SD, 12.9). Most of the patients had severe impairment of activities of daily living, as suggested by the mean BADLS of 43.6 (SD, 10.2).

**Table 1:** Characteristics of the sample (N = 242).

	N	Mean/Prop.	SD
DASS depression subscale		21.0	11.6
DASS anxiety subscale		20.2	10.9
Caregiver age, yrs		44.4	14.8
Caregiver sex (female)		0.75	0.43
Caregiver education, yrs		5.12	4.55
Caregiving duration, yrs		8.44	2.60
ZBI, total score		56.9	12.9
BADLS, total score		43.6	10.2
Patient age, yrs		86.5	8.85
Patient sex (female)		0.83	0.37
*Patient's relationship to caregiver*			
Grandparent	36	0.15	
Parent	181	0.75	
Spouse/other	25	0.10	
Fruit consumption freq. (d/wk)		2.54	2.35
*Fruit consumption, type*			
Avocado	70	0.29	
Citrus	16	0.07	
Jackfruit	15	0.06	
None	140	0.58	
Missing	1	0.004	
Vegetable consumption freq. (d/wk)		4.18	2.29
*Vegetable consumption, type*			
Green Leafy	93	0.38	
Root	11	0.05	
Other	19	0.08	
None	118	0.49	
Missing	1	.004	
Milk consumption freq. (d/wk)		0.65	1.58

Notes: BADLS, Bristol Activities of Daily Living Scale; DASS, Depression, Anxiety, and Stress Scales; SD, standard deviation; ZBI, Zarit Burden Interview

Consumption of fruits, vegetables and milk varied in this sample: fruit, 2.54 days per week (SD, 2.35; median, 2; interquartile range [IQR], 0-3); vegetables, 4.18 days per week (SD, 2.29; median, 4; IQR, 2-7); and milk, 0.65 days per week (SD, 1.58; median, 0; IQR, 0-0). Ninety-three (39%) participants consumed green leafy vegetables, 11 (5%) consumed root vegetables, and 19 (8%) consumed other vegetables. The most commonly reported fruit consumed was avocado (70 [29%]). There was one missing observation for type of fruit consumed and one missing observation for type of vegetable consumed.

### 3.2 Association between dietary diversity and depression symptom severity

To estimate the association between fruit and vegetable consumption and symptoms of depression, we fitted four multivariable regression models (Table 2). The first regression model included fruit types and their consumption frequency as explanatory variables while adjusting for covariates. Consumption of the different fruit types had statistically significant negative associations with depression symptom severity: avocado, b = -11.4 (95% confidence interval [CI], -14.2 to -8.66); citrus, b = -11.2 (95% CI, -14.3 to -8.13); and jackfruit, b = -14.2 (95% CI, -17.9 to -10.6). Other factors that correlated with depression were caregiver sex (female) and caregiving burden. This model explained 52% of the variation in depression symptom severity.

**Table 2:** Estimated associations between dietary diversity and depression symptom severity.

	Estimated regression coefficient (b)			
Variable	Model 1	Model 2	Model 3	Model 4
*Fruit consumption, type*				
Avocado	-11.4***			-0.77
Citrus	-11.2***			-0.74
Jackfruit	-14.2***			-4.68*
None	(base)			(base)
*Fruit consumption freq.*				
Infrequent (0-2 d/wk)	(base)			(base)
Frequent (3-7 d/wk)	-0.69			-0.06
*Vegetable consumption, type*				
Green Leafy		-15.6***		-14.1***
Root		-16.8***		-14.0***
Other		-16.4***		-14.8***
None		(base)		(base)
*Vegetable consumption freq.*				
Infrequent (0-4 d/wk)		(base)		(base)
Frequent (5-7 d/wk)		-1.84		-1.91*
*Milk consumption freq.*				
None			(base)	(base)
Some (1-7 d/wk)			-7.58***	-2.15*
Caregiver age, yrs	0.09	0.05	0.12*	0.04
Caregiver sex (female)	4.59**	2.57*	6.54***	2.28*
Caregiver education, yrs	0.19	0.05	0.21	0.09
ZBI, total score	0.18***	0.13***	0.25***	0.13***
BADLS, total score	0.11	0.03	0.19**	0.03
Patient age, yrs	-0.08	-0.06	-0.12	-0.06
Patient sex (female)	-0.56	-0.30	-0.64	-0.46
*Patient's relationshipto caregiver*				
Grandparent	(base)	(base)	(base)	(base)
Parent	-2.27	-2.62	-2.61	-2.18
Spouse/other	-2.84	-1.12	-3.35	-0.64
Constant	11.9	24.1***	2.03	24.6***

Notes: BADLS, Bristol Activities of Daily Living Scale; ZBI, Zarit Burden Interview; * p<0.05; ** p<0.01; *** p<0.001

The second regression model included vegetable types and their consumption frequency as explanatory variables while adjusting for covariates. Consumption of the different vegetable types had statistically significant negative associations with symptoms of depression: green leafy vegetables, b = -15.6 (95% CI, -18.1 to -13.0); root vegetables, b = -16.8 (95% CI, -20.2 to -13.3); and other vegetables, b = -16.4 (95% CI, -20.0 to -12.8). This model explained 66% of the variation in depression symptom severity.

The third regression model included the consumption frequency of milk as an explanatory variable while adjusting for covariates. Frequent milk consumption had a statistically significant negative association with depression symptom severity (b = -7.58; 95% CI, -10.2 to -4.93). This model explained 37% of the variation in depression.

In the final regression model for depression symptom severity, we included fruit, vegetable, and milk consumption together as explanatory variables while adjusting for covariates. In this analysis, we found that consumption of jackfruits, green leafy vegetables, root vegetables, other vegetables, frequent vegetable consumption, and frequent milk consumption all had a statistically significant negative association with symptoms of depression. This model explained 66% of the variance in depression symptom severity.

### 3.3 Association between dietary diversity and anxiety symptom severity

To estimate the association between fruit and vegetable consumption and symptoms of anxiety, we fitted four multivariable regression models (Table 3). The first regression model included fruit types and their consumption frequency as explanatory variables while adjusting for covariates. Consumption of the different fruit types had a statistically significant negative associations with anxiety symptom severity: avocado, b = -10.7; (95% confidence interval [CI], -13.3 to -8.18; citrus, b = -10.2 (95% CI, -13.0 to -7.47); and jackfruit, b = -12.4; (95% CI, -16.1 to -8.65). Other factors that correlated with anxiety were caregiver sex (female) and caregiving burden. This model explained 51% of the variation in anxiety symptom severity.

**Table 3:** Estimated associations between dietary diversity and anxiety symptom severity.

	Estimated regression coefficient (b)			
Variable	Model 1	Model 2	Model 3	Model 4
*Fruit consumption, type*				
Avocado	-10.7**			-1.47
Citrus	-10.2***			-1.13
Jackfruit	-12.4***			-3.83
None	(base)			(base)
*Fruit consumption freq.*				
Infrequent (0-2 d/wk)	(base)			(base)
Frequent (3-7 d/wk)	-0.02			0.60
*Vegetable consumption, type*				
Green Leafy		-13.8***		-12.2***
Root		-15.3***		-12.6***
Other		-14.6***		-12.7***
None		(base)		(base)
*Vegetable consumption freq.*				
Infrequent (0-4d/wk)		(base)		(base)
Frequent (5-7 d/wk)		-2.03*		-2.07*
*Milk consumption freq. (d/wk)*				
None			(base)	(base)
Some (1-7 d/wk)			-6.61***	-1.82
Caregiver age, yrs	0.10	0.06	0.12*	0.06
Caregiver sex (female)	4.37***	2.57*	6.04***	2.42*
Caregiver education, yrs	0.14	0.02	0.16	0.05
ZBI, total score	0.18***	0.13***	0.25***	0.13***
BADLS, total score	0.11*	0.05	0.19**	0.05
Patient age, yrs	-0.10	-0.09	-0.14	-0.09
Patient sex (female)	-0.09	0.17	-0.24	0.04
*Patient's relationshipto caregiver*				
Grandparent	(base)	(base)	(base)	(base)
Parent	-2.05	-2.18	-2.18	-2.00
Spouse/other	-4.03	-2.20	-4.21	-2.14
Constant	11.6	22.9***	2.90	22.8***

Notes: BADLS, Bristol Activities of Daily Living Scale; ZBI, Zarit Burden Interview; * p<0.05; ** p<0.01; *** p<0.001

The second regression model included vegetable types and their consumption frequency as explanatory variables while adjusting for covariates. Consumption of the different vegetable types and frequency of vegetable consumption had statistically significant negative associations with symptoms of anxiety: green leafy vegetables, b = -13.8 (95% CI, -16.2 to -11.4); root vegetables, b = -15.3 (95% CI, -18.85 to -11.7); other vegetables, b = -14.6 (95% CI, -17.8 to -11.3); and frequent consumption of vegetables, b = -2.03; (95% CI, -3.82 to -0.25). This model explained 64% of the variation in anxiety symptom severity.

The third regression model included the consumption frequency of milk as an explanatory variable while adjusting for covariates. Frequent milk consumption had a statistically significant negative association with anxiety symptom severity (b = -6.61; 95% CI, -9.08 to -4.14). This model explained 37% of the variation in anxiety symptom severity.

In the final regression model for anxiety symptom severity, we included fruit, vegetable, and milk consumption together as explanatory variables while adjusting for covariates. In this analysis, we found that consumption of green leafy vegetables, root vegetables, other vegetables, frequent vegetable consumption, and frequent milk consumption all had statistically significant negative associations with anxiety symptom severity. This model explained 65% of the variance in anxiety symptom severity.

### 3.4 Sensitivity analysis

We conducted an e-value analysis to determine the robustness of our findings to potentially unobserved confounding. In the multivariable regression model for depression symptom severity, the e-value associated with the point estimate for green leafy vegetable consumption was 4.61, and the e-value associated with the confidence interval was 3.44. In the multivariable regression model for anxiety symptom severity, the e-value associated with the point estimate was 4.34, and the e-value associated with the confidence interval was 3.23. These suggests that only very strong confounding would be sufficient to completely explain away the estimated associations between green leafy vegetable consumption and either depression or anxiety symptom severity. An unobserved confounder would need to have a strength of association exceeding 3-4, on the risk ratio scale, with both green leafy vegetable consumption and with depression (or anxiety) symptom severity in order to move our estimated regression coefficients to to have a confidence interval that included 0.

## 4 Discussion

This study sought to estimate the association between the consumption of different types of fruits and vegetables and symptoms of depression and anxiety in a sample of family caregivers of people living with dementia in southwestern Uganda. We found that the consumption of different categories of fruits and vegetables was negatively correlated with symptoms of both depression and anxiety. These findings are in line with previous studies that found an inverse association between vegetable consumption and symptoms of depression and anxiety (Altun, Brown, Szoeke, & Goodwill, 2019; Baharzadeh et al., 2018; Gibson-Smith et al., 2020; Głąbska, Guzek, Groele, & Gutkowska, 2020).

Also similar to our findings on the inverse association between fruit and vegetable consumption and symptoms of depression and anxiety, previous studies have found a dose-response relationship between consumption of fruits and vegetable and improvement in mental well-being and life satisfaction (Blanchflower, Oswald, & Stewart-Brown, 2013; Ocean, Howley, & Ensor, 2019; Peltzer & Pengpid, 2017; White, Horwath, & Conner, 2013). Our results are also consistent with previous studies that have found a correlation between better mental health and specific fruits and vegetables such as citrus fruits, carrots, cucumbers, and green leafy vegetables (Brookie et al., 2018; Conner, Brookie, Carr, Mainvil, & Vissers, 2017; Głąbska, Guzek, Groele, & Gutkowska., 2020).

Contrary to other studies that estimated an inverse association between fruit consumption and mental health problems in the general population (Adams & Colner, 2008; Allgöwer, Wardle, & Steptoe, 2001), we did not estimate a statistically significant correlation between fruit types and symptoms of anxiety after adjusting for other groups of vegetables. This discrepancy between our findings and previous studies can potentially be explained by the generally low levels of fruit consumption in this region of southwestern Uganda, which relies heavily on a starch-based diet anchored by the starchy banana *matoke*. In addition, whereas vegetables are readily available in this part of the country, many of these fruits are not locally grown and are typically more expensive than vegetables. Future work, including qualitative research, should attempt to better understand this null finding by conducting a more detailed examination of the consumption of fruits and vegetables in Ugandan communities.

Given the clinical and public health importance of fruit and vegetable consumption for mental well-being in the general population (Boehm et al., 2018; Gehlich et al., 2020; Głąbska et al., 2020; Sahraian, Ghanizadeh, & Kazemeini, 2015), our findings are particularly important for bridging the research gap in this area to lay the foundation for clinical and policy work. Future work, perhaps longitudinal studies, may focus on the interactions between consumption of different fruits and vegetables to tease out specific impacts of each category of fruit and vegetables. For caregivers of people with dementia specifically, our findings point to potential dietary interventions that can be used to enhance mental well-being in this population that is at high risk of mental health problems (Ainamani et al., 2020a).

Interpretation of our findings is subject to certain limitations. First, the sample was restricted to caregivers of people with ADRD and might not generalize to general population samples. A second important limitation of our study is the cross-sectional design, which does not allow for causal conclusions. Third, dietary intake was assessed retrospectively and may have been subject to misreporting or recall bias. The latter two limitations belong to a general class of concerns about confounding by unobserved variables: for example, poorer people may be less likely to consume fruits and vegetables, more likely to be food insecure, and more likely to report mental health problems (Lund et al., 2010; Tsai, Bangsberg, et al., 2012). Alternatively, people with memory problems may be more likely to under-report fruit and vegetable consumption and more likely to be depressed. However, our e-value analysis suggests that an unobserved confounder (such as socioeconomic status, food insecurity, or memory impairment) would need to have a very strong association with both fruit and vegetable consumption and with mental health problems in order to move our estimated regression coefficient and its confidence interval to include zero. Despite these limitations, our results suggest that fruits and vegetable consumption is negatively correlated with depressive and anxiety symptoms among the caregivers of people living with dementia.

**Abbreviations**
BADLS: Bristol Activities of Daily Living Scale
LAMICs: Low- and middle-income countries
ZBI: Zarit Burden Interview

## Consent for publication

Not applicable

## Availability of data and material

The data sets used and analyzed during the current study are available from the corresponding author on request.

## Ethics approval and consent to participate

Ethical approval for the study was obtained from the Mbarara University of Science and Technology. We also received clearance to conduct the study from the Uganda National Council for Science and Technology and the Research Secretariat in the President's office.

## Funding

HEA was supported through the Innovative Methods and Metrics for Agriculture and Nutrition Action (IMMANA) programme, led by the London School of Hygiene & Tropical Medicine (LSHTM). IMMANA is confounded with UKAid from the UK government and by the Bill & Melinda Gates Foundation (INV-002962/OPP1211308). Under the grant conditions of the foundation, a Creative Commons Attribution 4.0 Generic Lincense has already been assigned to the author Accepted Manunscripts version that might arise from this submission. ACT acknowledges salary support from the U.S. National Institutes of Health R01MH113494.

## Authors’ contributions

HEA participated in the conception and design of the study, collected the data, performed data analyses, interpreted the data, and drafted the manuscript. WMB participated in the conception of the study, supervised data collection, and revised the manuscript. GZR, ST, VM, and EMB participated in the conception of the study and revised the manuscript. ACT participated in the conception of the study, supervised data analysis, and provided substantial revision of the manuscript. All authors read and approved the final manuscript.

## Declaration of Competing Interest

The authors declare that they have no competing interests.
